# PHYSICAL ACTIVITY PROFILE AND PREFERENCE AMONG OLDER ADULT PATIENTS WITH CANCER: A CROSS-SECTIONAL STUDY

**DOI:** 10.1093/geroni/igad104.2538

**Published:** 2023-12-21

**Authors:** Donya Nemati, Tarah Ballinger

**Affiliations:** The Ohio state University, College of Nursing, Columbus, Ohio, United States; Indiana University School of Medicine, Indianapolis, Indiana, United States

## Abstract

Physical activity (PA) is beneficial for older adults (OAs) with cancer by improving physical function, mental health, and lowering the risk of recurrence in some cancer types. However, limited knowledge on OAs preferences may impede effective interventions. This study aimed to identify the PA profiles and preferences of OAs (age≥ 65) compared to younger adult (YA;18-64 years old) cancer patients. Data were collected from 372 adult patients with non-metastatic cancers visiting Indiana University Simon Comprehensive Cancer Center between July 2020-June 2021. Patients completed a cross-sectional survey comprising disease characteristics, PA habits, and PA preferences. A total of 132 OA patients (age 71 ± 5.2 years) were predominantly male (56.1%) and Caucasian (92.4%). Of these, 33.8% reported a decrease in PA after their cancer diagnosis. Fatigue (22%) and lack of motivation (16%) were the most reported PA barriers. While 43% of patients received exercise recommendations from their oncologists, 75% of those did not receive specific guidance. The majority of OAs preferred home-based workouts (65.9%) and exercise motivation packages (52.7%) over exercising at a cancer center facility (26%). Compared to YA patients (n=237), a smaller percentage of OAs (26.5% vs. 38.4%: p<.05) believed that PA could reduce the risk of cancer recurrence, and time constraints were less of a barrier for OAs (7.6% vs. 21.5%: p<.05). A home-based PA program that is supported with motivation packages may appeal to OAs with cancer. These findings are informative for designing tailored PA programs that suit the interests and preferences of OA patients with cancer.

